# Immunomagnetic separation of tumor initiating cells by screening two surface markers

**DOI:** 10.1038/srep40632

**Published:** 2017-01-11

**Authors:** Chen Sun, Yuan-Pang Hsieh, Sai Ma, Shuo Geng, Zhenning Cao, Liwu Li, Chang Lu

**Affiliations:** 1 Department of Biomedical Engineering and Mechanics, Virginia Tech, Blacksburg, Virginia 24061, USA; 2 Department of Chemical Engineering, Virginia Tech, Blacksburg, Virginia 24061, USA; 3 Department of Biological Sciences, Virginia Tech, Blacksburg, VA 24061, USA

## Abstract

Isolating tumor initiating cells (TICs) often requires screening of multiple surface markers, sometimes with opposite preferences. This creates a challenge for using bead-based immunomagnetic separation (IMS) that typically enriches cells based on one abundant marker. Here, we propose a new strategy that allows isolation of CD44^+^/CD24^−^ TICs by IMS involving both magnetic beads coated by anti-CD44 antibody and nonmagnetic beads coated by anti-CD24 antibody (referred to as two-bead IMS). Cells enriched with our approach showed significant enhancement in TIC marker expression (examined by flow cytometry) and improved tumorsphere formation efficiency. Our method will extend the application of IMS to cell subsets characterized by multiple markers.

There has been increasing proposition and observation that cancer growth is driven and sustained by tumor initiating cells (TICs, also known as cancer stem cells or CSCs) that are capable of self-renewal and aberrant differentiation^1^,^2^,^3^. Critical implications stem from the TIC model for understanding of cancer biology and, more importantly, design of new and more effective antitumor treatment^4^,^5^,^6^. TICs are commonly defined by a distinctive profile of surface markers and can be reproducibly isolated as a subset from tumor samples^7^,^8^,^9^. However, TIC detection and isolation present unique challenges due to their low frequency in most human tumors^2^,^9^,^10^,^11^. Currently, the most common method for TIC isolation is to use a fluorescence activated cell sorter (FACS) to sort out cells that present the desired surface profile after fluorescent labeling. FACS is an expensive instrument and typically only available at centralized facilities. FACS also requires complex optical and electronic systems that make it hard to implement it on a microfluidic platform.

Microfluidics provides a powerful platform for rare cell separation and analysis because of its ability of handling minute amounts of samples with high precision and integration^12^,^13^,^14^,^15^,^16^. TIC isolation based on physical properties of cells (e.g. dielectrophoretic response^17^ and deformability^18^) has been performed on microfluidic platforms. However, these methods do not directly screen surface markers and may not select the identical subset isolated by surface-marker-based methods. Immunomagnetic separation (IMS) combines high specificity of immunoassays and minimal invasiveness of magnetic force and is highly compatible with microfluidic platform. IMS is also compatible with handling a low number of cells (e.g. <10,000 cells), that would be a difficult task using FACS. Although IMS has been used for rare cell isolations^19^,^20^,^21^ (e.g. circulating tumor cells^22^,^23^,^24^, virus infected cells^25^,^26^, rare bacteria^27^,^28^, cancer cells^29^,^30^, etc.), no TIC isolation with IMS has been reported. Existing IMS methods sort cells based on a single surface marker that is highly expressed. In contrast, TICs are usually identified by multiple markers^7^,^8^,^9^ (such as CD44+/CD24^−^ population in breast cancer^11^,^31^,^32^,^33^, CD34^+^/CD38^−^ population in leukemia^34^, CD44^+^/α_2_ β_1_^hi^ /CD133^+^ population in prostate cancer^35^).

In this report, we demonstrate a new strategy for TIC isolation based on two markers of opposite selection criteria by using a combination of magnetic beads and nonmagnetic beads for IMS. In our method, CD44^+^/CD24^−^ cells were isolated from breast cancer cells (SUM149) by first mixing with anti-CD24-antibody-coated nonmagnetic beads before mixing with anti-CD44-antibody-coated magnetic beads. We then use a magnetic field to trap CD44^+^/CD24^−^ cells in a microfluidic channel in one step. Cells enriched with our two-bead IMS method showed high percentage of CD44^+^/CD24^−^ population (41.7% compared to 10.3% before separation, and 19.4% for using only anti-CD44-coated magnetic beads), as showed by flow cytometry analysis. Cells sorted by two-bead IMS yielded 1.62% tumorsphere formation compared to 1.16% with one-bead IMS using only CD44^+^ criterion, and 0.62% before separation, when the tumorsphere cultures started with same initial cell number. Combined with the capability of a microfluidic platform for handling a small number of cells, we envision our technology has the potential to extend the application of IMS to highly specific TIC enrichment from scarce samples.

## Results and Discussion

**Figure 1:**
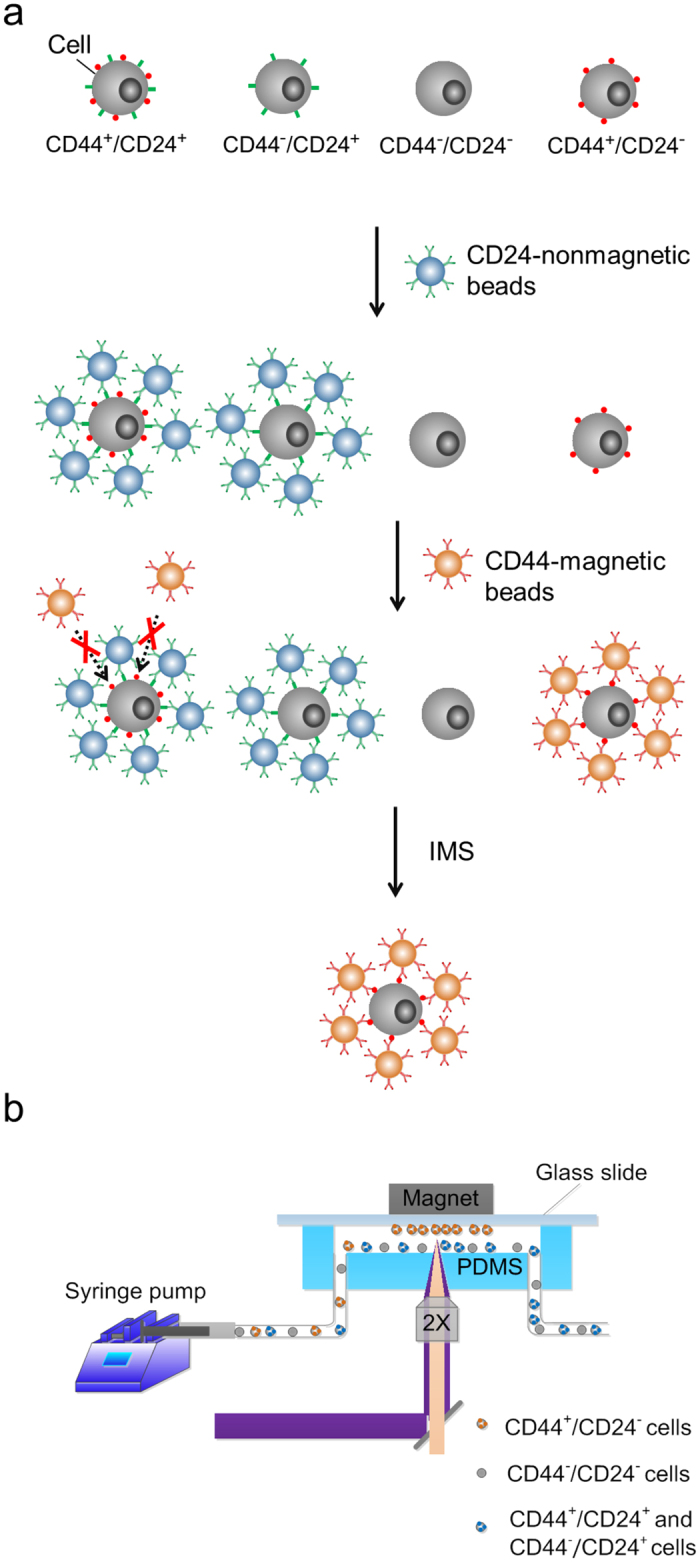
IMS of CD44^+^/CD24^−^ cells using two types of beads. (**a**) Schematic for sequential use of CD24-antibody-coated nonmagnetic beads and CD44-antibody-coated magnetic beads to magnetically label CD44^+^/CD24^−^ cells. (**b**) The experimental setup for immunomagnetic separation in a microfluidic channel.

Breast cancer cell line SUM149 cells were labelled sequentially with anti-CD24-coated nonmagnetic beads and anti-CD44-coated magnetic beads, as shown in Fig. 1a. Nonmagnetic polystyrene beads (~4.95 *μ*m in diameter, Bangs Laboratories) were functionalized with anti-CD24 antibody (referred to as CD24-nonmagnetic beads), and superparamagnetic polystyrene beads (Dynabeads, 4.5 *μ*m in diameter, Life technologies) were functionalized with anti-CD44 antibody (referred to as CD44-magnetic beads) via streptavidin-biotin interactions. The linkage between magnetic bead and streptavidin is a cleavable DNA linker that allows whole cell separation from the beads when cell culturing is desired in the downstream. SUM149 cells were firstly mixed with CD24-nonmagnetic beads. Cells with a high expression level of CD24 antigen (CD24^+^ cells) were conjugated with the beads, while CD24- cells remained unoccupied. In the second mixing, CD44-magnetic beads could only bind to CD44^+^/CD24^−^ cells (i.e. they could not bind to CD44^+^/CD24^+^ due to the spatial hindrance from the bound CD24-nonmagnetic beads from the previous step). The net result was that only CD44^+^/CD24^−^ cells were magnetically labeled. Cell/bead complexes were then flown into a microfluidic device for magnetic isolation, as shown in Fig. 1b. Magnetically captured cells were eluted from the channel after removing the magnet and collecting at the outlet. About 2–4% of starting cells were recovered after all steps. Some of this loss was due to removal of beads from cells which may not be entirely necessary, depending on what type of downstream analysis follows (e.g. analysis of genomic DNA can be performed by lysing and releasing chromosome without removing the beads).

**Figure 2:**
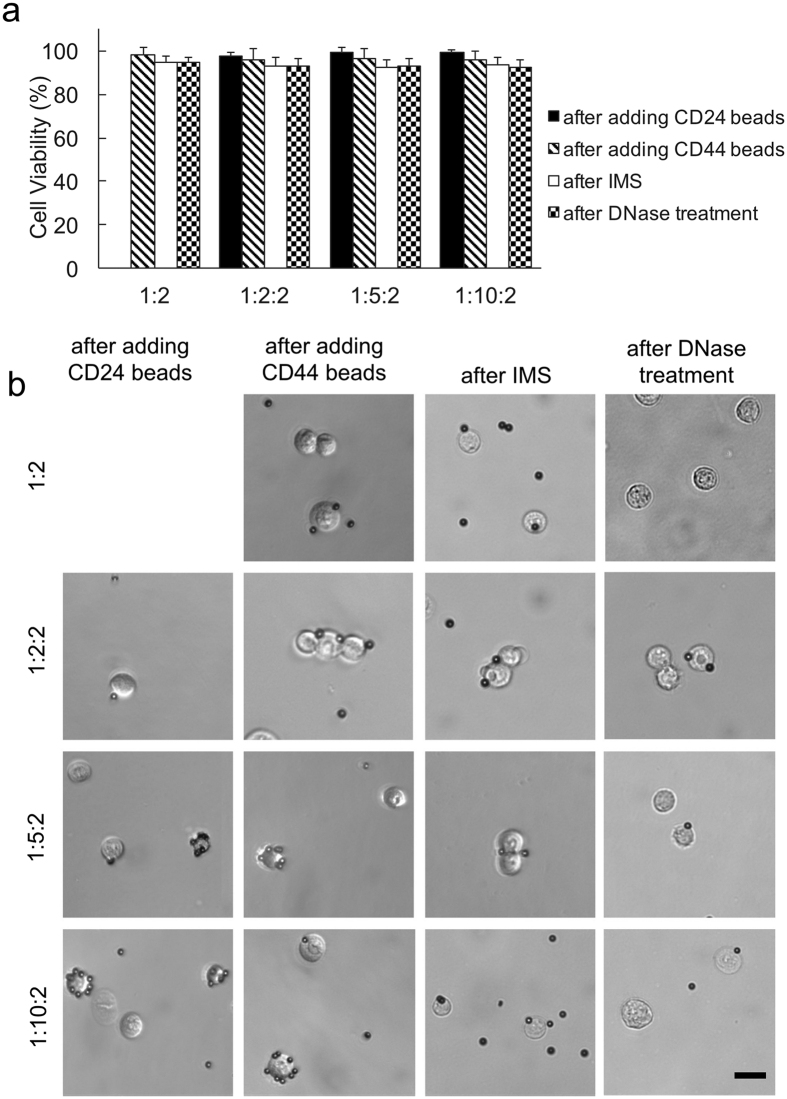
The cell viability and image after various steps. (**a**) Cell viability after each step in the protocol; (**b**) Representative images of cell/bead complexes after each step. Various bead mixing conditions were used: “1:2”: cells and CD44-magnetic beads mixed with a ratio of 1:2 (cell number/bead number); “1:2:2”: cells mixed with CD24-nonmagnetic with a ratio of 1:2 and then with CD44-magnetic beads with a ratio of 1:2; “1:5:2”: cells mixed with CD24-nonmagnetic with a ratio of 1:5 and then with CD44-magnetic beads with a ratio of 1:2; “1:10:2”: cells mixed with CD24-nonmagnetic with a ratio of 1:10 and then with CD44-magnetic beads with a ratio of 1:2. Scale bars: 20 *μ*m.

There was no significant loss of cell viability after each experimental treatment (Fig. 2a). We examined 4 bead mixing conditions: one-bead/1:2 (cells and CD44-magnetic beads were mixed with a ratio of 1:2), two-bead/1:2:2 (cells were mixed with CD24-nonmagnetic with a ratio of 1:2 and then with CD44-magnetic beads with a ratio of 1:2), two-bead/1:5:2, and two-bead/1:10:2. Figure 2b shows the representative images of cell/bead complexes after each step. There was substantial blocking of cell surface area after CD24-nonmagnetic beads were added (the column of images labeled as “after adding CD24 beads”) and the degree of cell surface coverage increased when the amount of CD24 beads increased from 1:5:2 to 1:10:2. These heavily covered cells (mostly by CD24-nonmagnetic beads) were not present after IMS.

**Figure 3:**
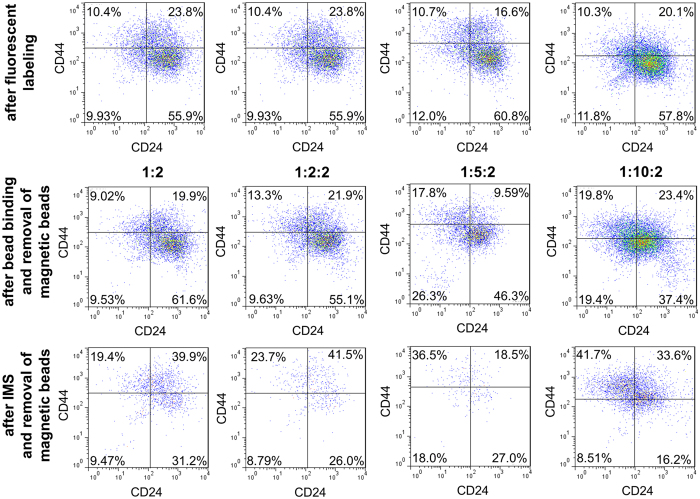
Flow cytometry data show that two-bead IMS method yielded high percentage of CD44^+^/CD24^−^ population. Color density plots of CD44 and CD24 expression on SUM 149 cells after fluorescent labeling (top row), after bead binding and removal of magnetic beads (middle row) and after IMS and removal of magnetic beads (bottom row) under various bead mixing conditions.

We examined the surface TIC markers, CD44 and CD24, before and after our IMS using flow cytometry in order to confirm the enrichment by our IMS method (Fig. 3). Quantitative measurement using flow cytometry requires setting up a standard to compensate shifts in the relative fluorescence intensity among different set of experiments (sometimes taken on different days). The color density plots in the top row of Fig. 3 show the data taken using SUM149 cells after immunofluorescent labeling of CD44 and CD24. These plots serve as the standard for dividing 4 quadrants for each set of data. These standard plots were all obtained using labeled SUM149 cells on the day when the set of flow cytometry data (i.e. the other plots in the same column in Fig. 3) were taken. We divided the quadrants in these plots in such a way that all 4 quadrants contain roughly the same percentages of the cell population. We then used the same subdivisions for the rest of the plots in the same data set. The middle row of Fig. 3 shows the color density plots of cells after mixing with and binding to beads under various conditions and then removing CD44 beads by DNase cleavage. We tested using one-bead/1:2, two-bead/1:2:2, two-bead/1:5:2, and two-bead/1:10:2. Before examination by flow cytometry, CD44-magnetic beads were released from the cell surface via DNase cleavage of the link between bead surface and streptavidin. However, nonmagnetic beads remained on cell surface. The cell populations examined included increasing amount of cell/bead complexes when the amount of CD24-nonmagnetic beads used increased. This affected the fluorescence intensity of the cell population observed (e.g. the upper left quadrant, i.e. the CD44^+^/CD24^−^ subpopulation, in all the plots in the middle row of Fig. 3 exhibit larger percentages than their standard plots in the top row.). In the bottom row of Fig. 3, after IMS and removal of magnetic beads from the cell surface, flow cytometry was used to examine the isolated cells under each condition. The percentage of the upper left CD44^+^/CD24^−^ quadrant all increased significantly after the IMS (with either one-bead or two-bead procedure). With one-bead IMS, we were only selecting cells base on the abundance of CD44 and we increased the upper left quadrant percentage by 10.4% (from 9.0% to 19.4% as we used the middle row flow cytometry data as the reference). The enrichment increase was similar when we added 2X CD24-nonmagnetic beads (in two-bead/1:2:2). When we further increased the amount of CD24-nonmagnetic beads in two-bead/1:5:2 and two-bead/1:10:2, the use of more CD24-nonmagnetic beads removed more CD24^+^ cells and the upper left quadrant experienced a more substantial enrichment by 18.7% (from 17.8 to 36.5%) and 21.9% (from 19.8 to 41.7%) due to IMS, respectively. This confirmed that the use of CD24-nonmagnetic beads was a critical piece of our cell isolation strategy, as depicted in Fig. 1.

**Figure 4:**
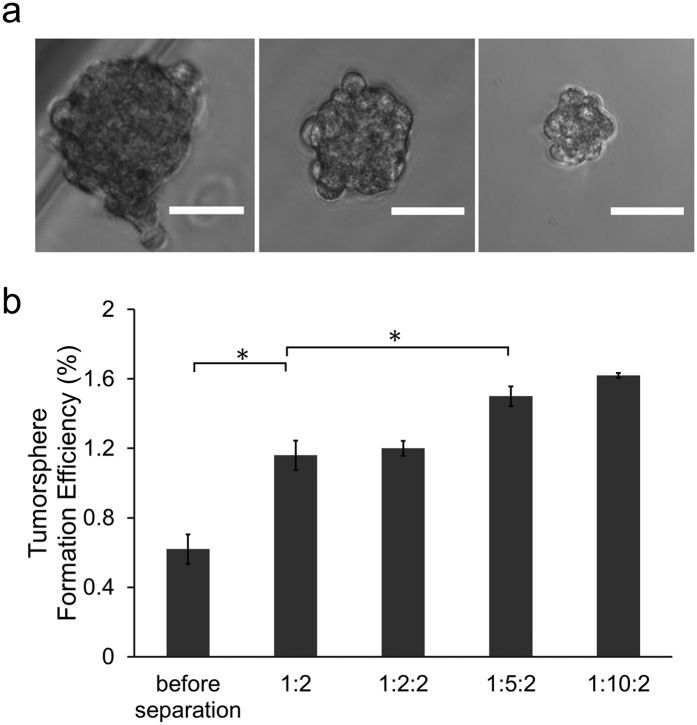
Cells separated with our “two-bead” IMS method showed high tumorsphere formation efficiency. (**a**) Representative images of tumorspheres formed by SUM 149 cells. Scale bars: 50 *μ*m. (**b**) Tumorsphere formation efficiency for cell populations produced under various separation conditions. *P < 0.05.

The most important and characteristic feature of TICs is their ability to drive the formation of spheroids, known as tumorspheres^36^,^37^. We assessed tumor initiating ability of cells before and after isolation using one-bead or two-bead IMS approaches. Cells were seeded at 2,500 cells per well in low-adhesion 6-well plates and the morphology and the number of tumorspheres were evaluated 7 days later. The tumorspheres formed had a size range of 40–120 μm (Fig. 4a). Approximate 0.62% of unsorted SUM149 cells formed tumorspheres (Fig. 4b) and this percentage matches the range of tumorsphere formation efficiency reported in the literature^18^,^38^,^39^,^40^. Tumorsphere formation efficiency was increased to 1.16% when we applied one-bead/1:2 IMS and used the isolated cells. In comparison, there was no significant further improvement in the tumorsphere formation efficiency using two-bead/1:2:2 IMS enrichment and this is consistent with the flow cytometry results in Fig. 3. The percentage of tumorsphere-forming cells with two-bead/1:5:2 IMS increased to 1.50% (i.e. a factor of 2.4 increase comparted to cells without sorting), providing justification for the superiority of two-bead IMS method over one-bead isolation for TIC enrichment. The tumorsphere formation efficiency further increased to1.62% when the sphere culture started from cells sorted by two-bead/1:10:2. This number is comparable to the result generated by FACS sorting^18^,^38^,^39^,^40^. All these results are consistent with the results by flow cytometry (Fig. 3).

## Summary

We demonstrate a new strategy that allows isolation of TICs based on two markers of opposite preferences using magnetic and nonmagnetic beads that are coated by different antibodies. We show that cells enriched by our approach exhibit desired profiles in TIC markers (CD44^+^/CD24^−^). Tumorsphere formation efficiency of cells after two-bead IMS is significantly higher than cells before separation or after one-bead IMS. Compared to competing technologies such as FACS, our two-bead IMS does not require costly instruments and is particularly suitable for processing samples containing a low number of cells. The actual operational conditions of two-bead IMS will need to be adjusted based on the needs and goals of the research. Our method is not limited to the microfluidic platform used in the demonstration. The same principle can be applied using typical apparatus used in IMS.

## Methods

### Cell culture

SUM149 cells (a human breast cancer cell line) were grown in Ham’s F-12K medium (Life Technologies) supplemented with 5% fetal bovine serum (FBS) (Atlanta Biologicals), 5 *μ*g/ml insulin (Gibco), 1 *μ*g/ml hydrocortisone (Stemcell Technologies), and 1% penicillin/streptomycin (Invitrogen) in a humidified atmosphere of 5% CO_2_ at 37 °C. Cells were subcultured every 2–3 days at a ratio of 1:10 to maintain their exponential growth phase. Cells were collected in the culture medium after detaching from the flask substrate with trypsin-EDTA (Sigma).

### Labeling by beads

CD44-magnetic beads (i.e. magnetic beads coated with anti-CD44) were obtained by mixing 10 *μ*l streptavidin-coated superparamagnetic polystyrene beads (4 × 10^8^ beads/ml, 4.5 *μ*m diameter, Dynabeads, CELLection Biotin Binder Kit, Life technologies) with 1 *μ*g biotinylated anti-CD44 antibody (human, Clone DB105, Miltenyi Biotec) in 1 ml PBS and incubating overnight at 4 °C under gentle rotation (24 rpm). CD24-nonmagnetic beads were produced by mixing 30 *μ*l streptavidin-coated polystyrene beads (1.4 × 10^8^ beads/ml, 4.95 *μ*m in diameter, Bangs Laboratories, Inc.) with 1 *μ*g biotinylated anti-CD24 antibody (human, Clone 32D12, Miltenyi Biotec) in 1 ml PBS and incubating overnight at 4 °C under gentle rotation (24 rpm). The tube containing CD44-magnetic beads was then placed in magnet (Dynamag-5, Life technologies) for 1 min. The supernatant was then discarded to remove excessive anti-CD44 antibody. The suspension containing CD24-nonmagnetic beads was centrifuged at 300 g for 5 min before the supernatant was discarded to remove excessive anti-CD24 antibody. 1 ml SUM149 cells in PBS were added to the CD24-nonmagnetic beads (at a ratio of 1:2, 1:5 or 1:10, cell number/bead number) and mixed well by pipetting. The mixture was incubated at 4 °C for 1 h under rotation at 24 rpm. Then CD44-magnetic beads (at a ratio of 1:2, cell number/bead number) were added and mixed at 4 °C for 1 h with rotation at 24 rpm. After the reaction, cell-bead complexes were then centrifuged and resuspended in 0.6 ml PBS containing 7 wt% dextran (Sigma-Aldrich, added to match the density of buffer with that of cells to prevent cell settling due to gravity)^41^,^42^.

### Microfluidic chip fabrication

A microfluidic channel (with dimensions of 2.4 mm (W) × 0.2 mm (D) × 10 mm (L)) was fabricated using standard soft lithography^43^,^44^. Briefly, a photomask with microscale pattern designed by FreeHand MX (Macromedia) was printed on transparencies (Infinity Graphics) at a resolution of 5,080 dpi. The features on the photomask were transferred to a 3 inch silicon wafer by spin-coating a layer of ~ 200 *μ*m thick photoresist SU8 2125 (Clariant) followed by UV exposure and development. Polydimethylsiloxane (PDMS, General Electric Silicones RTV 615, MG chemicals) prepolymer mixture at a mass ratio of A:B = 10:1 was poured onto the silicon wafer to form a ~5 mm thick layer and cured in a 80 °C oven for 1 h. PDMS replica was then peel off and drilled for inlets and outlets before binding to a pre-cleaned glass by plasma treatment (Harrick Plasma). The assembled chip was baked at 80 °C for 1 h to ensure strong bonding between PDMS and glass.

### Cell separation on the microfluidic chip

The experimental setup used for microfluidic immunomagnetic separation is shown in Fig. 1b. 1% BSA in PBS was used to pretreat the microfluidic channel for 1 h at 37 °C to reduce nonspecific adhesion of cells and beads to the channel walls^45^. The glass side of the microfluidic device was facing up and a NdFeB magnet with maximum energy product of 42 MGOe (1/4″ × 1/4″ × 1/4″, K&J Magnetics) was placed on the glass substrate (~1 mm thickness). This setup also kept the magnet out of the observation path, so that the separation process could be monitored with an inverted fluorescence microscope (IX-83, Olympus). Cell/bead mixture were flowed through the microfluidic channel at a constant rate of 57.6 *μ*l/min sustained by a syringe pump (Harvard Apparatus) for separation. After the flow of the sample, we flowed PBS buffer for 1 min at 57.6 *μ*l/min to remove nonspecific adsorption. Magnetically captured cells were eluted at the same flow rate from the channel after removing the magnet and collected at the outlet. The separated cells (in the form of cell-bead complexes) were centrifuged and resuspended in 400 *μ*l culture media containing 0.8 *μ*l DNase I (CELLection Biotin Binder Kit, Life technologies), and incubated at 37 °C for 20 min with 600 rpm shaking. This step broke the DNA linker between bead surface and streptavidin to release magnetic beads (CD44 Beads) from cell surface. The magnetic beads were then removed from the suspension by a magnet (Dynamag-5, Life technologies). After each step, cell viability was examined by trypan blue (Thermo Fisher Scientific) staining.

### Flow cytometry

Cells (or cells with bound beads) were suspended in 100 *μ*l cold (4 °C) stain buffer (PBS, 2% FBS, 0.1% NaN_3_) for staining by fluorescently conjugated antibodies. 2 μl FITC anti-human CD44 antibody (Clone G44-26, BD Biosciences) and 2 μl PE anti-human CD24 antibody (Clone ML5, BD Biosciences) were added to the cell suspension according to manufacturer instructions and incubated for 45 min on ice, protected from light. After staining, the cells were washed twice with 1 ml stain buffer and kept in 400 *μ*l stain buffer on ice until analysis by FACS-Canto-II (BD Biosciences).

### Tumorsphere formation assay

Cells were plated at 2,500 cells per well in 6-well low-adhesion plates (Cat. no. 3471, Corning) with each well containing 3 ml complete Mammocult media (Mammocult basal media supplemented with 10% Mammocult proliferation supplement, 4 *μ*g/ml heparin, 0.48 *μ*g/ml hydrocortisone (Stemcell Technologies), and 1.5% penicillin/streptomycin (Invitrogen)), and incubated in a humidified atmosphere of 5% CO_2_ at 37 °C. After 7 d incubation, tumorspheres (size ≥ 40 *μ*m) formed per well were determined by visual counting under a microscope with 10X dry objective (Olympus IX-83). Tumorsphere formation efficiency was calculated as the number of tumorspheres divided by the initial number of cells seeded (2,500 cells). Images of tumorspheres were taken using an Olympus IX-83 inverted microscope with 20X dry objective.

## Additional Information

**How to cite this article**: Sun, C. *et al*. Immunomagnetic separation of tumor initiating cells by screening two surface markers. *Sci. Rep.* **7**, 40632; doi: 10.1038/srep40632 (2017).

**Publisher's note:** Springer Nature remains neutral with regard to jurisdictional claims in published maps and institutional affiliations.
